# Factors affecting the relationship between ionized and corrected calcium levels in peritoneal dialysis patients: a retrospective cross-sectional study

**DOI:** 10.1186/s12882-020-02033-y

**Published:** 2020-08-26

**Authors:** Masamitsu Morishita, Yukio Maruyama, Masatsugu Nakao, Nanae Matsuo, Yudo Tanno, Ichiro Ohkido, Masato Ikeda, Takashi Yokoo

**Affiliations:** Division of Nephrology and Hypertension, Department of Internal Medicine, The Jikei University School of Medicine, 3-25-8 Nishi-shinbashi, Minato-ku, Tokyo, 105-8461 Japan

**Keywords:** Peritoneal dialysis, Chronic kidney disease-mineral and bone disorder, Corrected calcium, Ionized calcium, Acid–base equilibrium, Residual renal function

## Abstract

**Background:**

Chronic kidney disease-mineral and bone disorder (CKD-MBD) management in patients with end-stage renal disease is important owing to the risk of cardiovascular diseases. In clinical practice, we manage patients not by monitoring the levels of biologically active ionized calcium (iCa) but by monitoring total serum calcium or corrected calcium (cCa). We previously reported that iCa/cCa ratio was different between patients with hemodialysis and those with peritoneal dialysis (PD). In PD patients, several factors are expected to affect iCa/cCa ratio. Therefore, modifying the strategy to achieve better CKD-MBD management might be necessary; however, no reports have studied this to date. Therefore, we investigated the factors influencing iCa/cCa ratio in PD patients.

**Methods:**

This retrospective cross-sectional study examined background and laboratory data, including iCa, collected at routine outpatient visits. The patients were divided into the first, second, and third tertile of iCa/cCa ratio groups to compare patient background and laboratory data. Multiple regression analysis was used to investigate the factors influencing iCa/cCa ratio. We used multiple imputation to deal with missing covariate data.

**Results:**

In total, 169 PD patients were enrolled. In PD patients with lower iCa/cCa ratio, PD duration was longer and pH was higher. Urine volume and weekly renal Kt/V were lower in the patients with lower iCa/cCa ratio than in those with higher iCa/cCa ratio. iCa/cCa ratio and weekly renal Kt/V were directly correlated (*r =* 0.41, *p* < 0.01), and weekly renal Kt/V and pH were independent factors affecting iCa/cCa ratio (t = 2.86, *p* < 0.01 and t = − 5.42, *p <* 0.01, respectively).

**Conclusions:**

iCa levels were lower in PD patients with lower residual renal function (RRF) even though their cCa levels were equal to those with maintained RRF, warranting caution in the assessment and management of CKD-MBD in PD patients.

## Background

Cardiovascular diseases are a major cause of death in dialysis patients [1, 2], and chronic kidney disease-mineral and bone disorder (CKD-MBD) is an important risk factor of dialysis [3, 4]. It has been reported that the management of CKD-MBD is crucial not only for dialysis patients but also for non-dialysis patients [5]. Therefore, the diagnosis and treatment guidelines of CKD-MBD were created with inputs from several countries and regions, including Japan, to establish the recommended levels of calcium (Ca), phosphorus (P), and parathyroid hormone (PTH) with the goal of the appropriate management of CKD-MBD [6–8].

Ionized Ca (iCa) is the biologically active form of Ca in the blood. However, considering its inconvenience and resultant high cost, there are few studies that have measured serum iCa levels in clinical settings [9]. Therefore, many guidelines do not list the recommended levels of iCa, but list those of target total serum Ca or corrected Ca (cCa) levels accounting for total serum Ca and serum albumin (Alb) levels [6–8]. Approximately 50% of serum Ca is present in the form of iCa, approximately 45% of which binds to proteins such as Alb, while the remaining 5% binds to acid to exist as Ca salts. Because blood iCa competes with hydrogen ions as it binds to Alb, it can be influenced by the acid–base equilibrium. During acidemia, in which the hydrogen ion concentration is increased, the quantity of iCa that binds to Alb decreases, resulting in higher blood iCa levels, while a lower iCa level is observed during alkalemia [10, 11].

Compared with HD patients, PD patients tend to be under metabolic alkalosis status owing to the characteristics of alkalized PD dialysate [12]. We previously reported that the acid–base equilibrium of peritoneal dialysis (PD) patients differed from that of hemodialysis (HD) patients; PD patients have a lower iCa/cCa ratio and tend to have lower iCa concentrations than their HD counterparts with the same cCa levels [13]. Therefore, we reported that managing Ca levels based on cCa levels requires caution. Moreover, we compared intact PTH (iPTH) levels in PD and HD patients with equal cCa levels and found that PD patients showed higher iPTH levels than HD patients [13].

In many regions, there are more HD patients than PD patients, and the importance of the management of CKD-MBD in HD patients has been reported. However, CKD-MBD is listed as an important complication of PD as well [14, 15]. There are several reports related to the importance of residual renal function (RRF) in managing CKD-MBD in PD patients [16–18]. The RRF of PD patients and the quantities of dialysis fluid used according to their RRF are predicted to affect their acid–base equilibria. Accordingly, differences in acid–base equilibrium among PD patients are predicted to affect the relationship between iCa and cCa levels (i.e., iCa/cCa ratio) and its management; therefore, uniform cCa evaluation in PD patients could result in imprecise iCa evaluation, and its correction could not only induce the modification of the CKD-MBD treatment strategy but also reduce the risk of cardiovascular diseases, the chief outcome of CKD-MBD, in PD patients. However, there are almost no reports that have actually tested this hypothesis. Therefore, the aim of this study was to investigate the factors affecting the relationship between iCa and cCa levels (iCa/cCa ratio) in PD patients.

## Methods

### Patients

This retrospective cross-sectional study enrolled stable patients with end-stage renal disease who were undergoing PD at Jikei University Hospital (Tokyo, Japan), Jikei University Kashiwa Hospital (Chiba, Japan), and Jikei University Katsushika Medical Center (Tokyo, Japan). They were over 20 years of age and were receiving PD treatments for more than 3 months. The exclusion criteria were as follows: ongoing combined therapy with PD and HD (6-day PD and one HD session per week) and those with the presence of acute infectious diseases, including PD-related peritonitis. Some of the patients from our previous report were also included in this study [13]. The primary objective of this study was to investigate the iCa/cCa ratio. The study protocol was approved by the Ethics Committee of Jikei University Hospital (approval number: 30–2,959,316). Written informed consent was waived owing to the non-interventional and retrospective nature of the study. Instead, all participants were provided a means to opt out of the study.

### Data collection

Patient data were obtained from April 2017 to August 2018. All blood samples, including those for blood gas analysis, were collected from the vein during regular outpatient visits. Samples were transported to the laboratory and analyzed immediately after collection. Serum iCa levels and pH were analyzed using the ABL 800 system (Radiometer, Copenhagen, Denmark) with an ion-selective electrode and pH electrode, respectively. Serum iPTH levels were assessed using the COBAS8000 (Roche Diagnostics GmbH, Manheim, Germany) via electrochemiluminescence immunoassay. Serum total Ca levels were measured using the TBA-2000 (Toshiba, Tokyo, Japan) with chlorophosphonazo III. Other biochemical parameters, including values of hemoglobin, creatinine, albumin, phosphorus, and C-reactive protein, were measured by standard laboratory techniques. We measured 24-h urine volume and examined weekly renal Kt/V, which were used as surrogate markers for RRF in this study, every 6 months as a periodical test. Blood and urine samples were collected and all biochemical parameters were measured simultaneously. Patients’ clinical information, including sex, age, PD duration, body mass index (BMI), underlying disease, comorbidity, PD prescription, and medication, were also obtained during regular outpatient visits by reviewing their medical records.

Weekly renal Kt/V was calculated using the formula recommended by the Japanese Guidelines for Peritoneal Dialysis [19], which uses Watson’s body fluid volume calculation [20].

Weekly renal Kt/V = {[urine urea nitrogen (mg/dL) × urine volume (mL/day)]/[serum urea nitrogen (mg/dL) × body fluid volume (L)]} × 7/1000.

Body fluid volume for males (mL) = 107.4 × height (cm) + 336.2 × BW (kg) + 2447–95.16 × age.

Body fluid volume for females (mL) = 106.9 × height (cm) + 246.6 × BW (kg) − 2097.

Albumin-corrected Ca levels were estimated using the Payne’s formula recommended in the Japanese Guidelines for CKD-MBD [8]: cCa = total serum Ca + [4 − serum Alb (g/dL)]. Serum albumin levels of ≥4.0 g/dL indicate that cCa level is equal to the total serum Ca level.

### Statistical analysis

Data were statistically analyzed using STATA version 16.0 (STATA Corporation, College Station, TX, USA). The patients were divided into the first, second, and third tertile of the iCa/cCa ratio groups. Patient characteristics are presented as mean ± standard deviation for normally distributed continuous variables, median and interquartile range for skewed continuous variables, and percentage for categorical variables. Differences among the three groups according to iCa/cCa ratio were analyzed using one-way analysis of variance or the non-parametric Kruskal–Wallis test as appropriate. The chi-squared test or Fisher’s exact test was used to compare nominal variables. The relationship between iCa/cCa ratio and weekly renal Kt/V in all patients was evaluated via linear regression analysis. We also divided the patients into higher or lower RRF group with the median weekly renal Kt/V of 0.46, and the relationship between the iCa and cCa levels of both groups were evaluated via linear regression analysis because we hypothesized that RRF has an impact on the iCa/cCa ratio in PD patients. Multiple regression analysis was used to evaluate the independent factors affecting iCa/cCa ratio. In this multivariate analysis, covariates were PD duration, weekly renal Kt/V, urinary volume, pH, hemoglobin levels, creatinine levels, phosphate levels, and dialysate volume. Multiple imputation was performed in multiple regression analysis. The missing values of all covariates were imputed by assuming that data were missing at random with 20 imputations. PD duration, weekly renal Kt/V, urinary volume, and dialysate volume were remarkably skewed and log-transformed to normalize the distribution before multiple imputation. *P* values of < 0.05 were considered statistically significant.

## Results

### Baseline characteristics

**Fig. 1:**
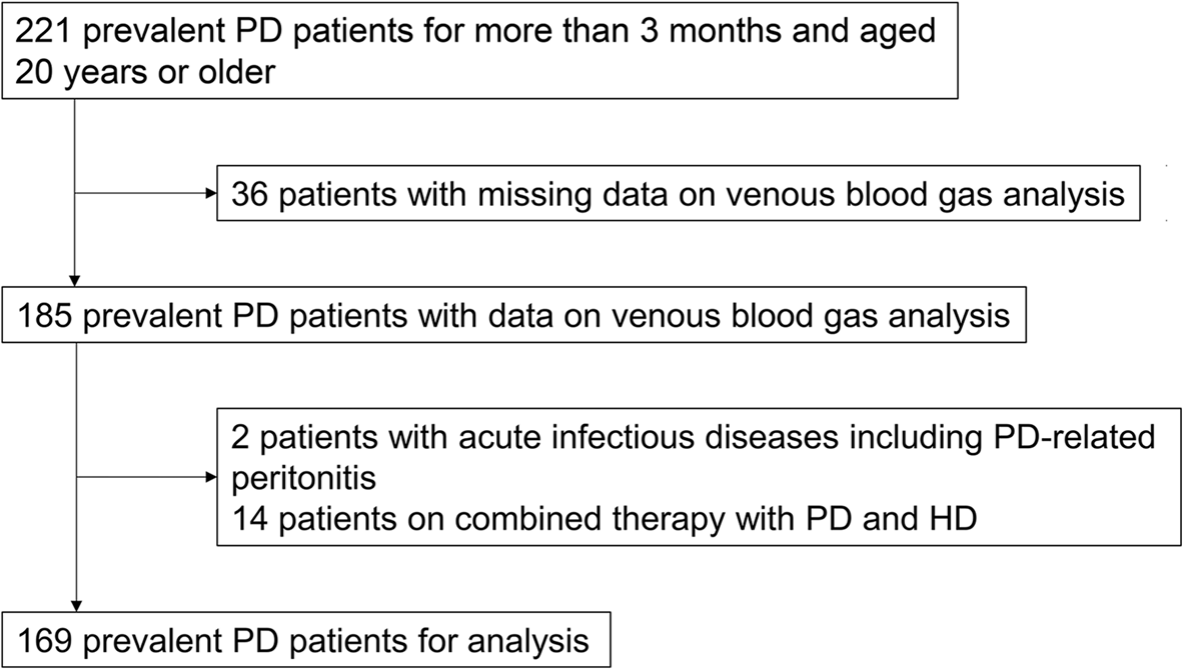
Patient selection flowchart. Abbreviations: PD, peritoneal dialysis; HD, hemodialysis

Figure 1 summarizes the process of patient selection. The number of patients enrolled initially was 221. After excluding patients with missing data on venous blood gas analysis and acute infectious diseases including PD-related peritonitis as well as patients on combined therapy with PD and HD, 169 patients were enrolled in this study.

The baseline characteristics of all patients are summarized in Table 1. Patients were divided into the first, second, and third tertile of the iCa/cCa ratio groups, and their cutoff values were 47.0 and 49.0%.

**Table 1:** Baseline characteristics of the 169 PD patients in our study cohort

Variable	No. of Missing Value (%)	Whole patients	iCa/cCa ratio			*P* value
			< 47.0%	47.0 to < 49.0%	≥49.0%	
Number	0 (0)	169	56 (33%)	54 (32%)	59 (35%)	
Male [%]	0 (0)	124 (73%)	40 (71%)	37 (69%)	47 (80%)	0.38
Age [y]	0 (0)	62 ± 13	63 ± 13	61 ± 14	61 ± 14	0.56
PD duration [months]	0 (0)	24 (11–50)	32 (12–74)	24 (12–44)	18 (9–40)	0.04
BMI [kg/m^2^]	0 (0)	23.7 ± 4.3	22.9 ± 4.1	23.8 ± 4.8	24.5 ± 4.0	0.14
Underlying disease						0.89
CGN [%]	0 (0)	50 (30%)	16 (28%)	18 (33%)	16 (27%)	
Diabetes [%]	0 (0)	61 (36%)	21 (38%)	21 (39%)	19 (32%)	
Nephrosclerosis [%]	0 (0)	29 (17%)	11 (20%)	6 (11%)	12 (20%)	
PCKD [%]	0 (0)	6 (4%)	1 (2%)	2 (4%)	3 (5%)	
Others or unknown [%]	0 (0)	23 (14%)	7 (13%)	7 (13%)	9 (15%)	
Comorbidity						
CVD [%]	0 (0)	52 (31%)	19 (34%)	22 (41%)	11 (19%)	0.03
Residual renal function						
Urine volume [ml]	25 (14.8)	700 (300–1500)	500 (200–1080)	600 (190–1420)	900 (540–1730)	0.01
Weekly renal Kt/V	49 (29.0)	0.46 (0.14–0.83)	0.25 (0.09–0.61)	0.43 (0.12–0.73)	0.66 (0.40–1.00)	< 0.01
PD solution						
Dialysate Volume [L]	0 (0)	5.8 ± 1.8	6.3 ± 1.5	5.7 ± 1.9	5.5 ± 1.9	0.045
Medication						
Cinacalcet [%]	0 (0)	47 (28%)	23 (41%)	13 (24%)	11 (19%)	0.02
Vitamin D [%]	0 (0)	91 (54%)	27 (48%)	29 (54%)	35 (59%)	0.49
Ca-containing P binder [%]	0 (0)	47 (28%)	23 (41%)	10 (19%)	14 (24%)	0.02
Ca-free P binder [%]	0 (0)	86 (51%)	26 (46%)	37 (69%)	23 (39%)	< 0.01
Laboratory data						
pH	0 (0)	7.34 ± 0.04	7.366 ± 0.033	7.339 ± 0.005	7.328 ± 0.005	< 0.01
Bicarbonate [mmol/L]	0 (0)	24.8 ± 2.5	25.3 ± 2.5	24.7 ± 2.3	24.3 ± 2.5	0.08
Hemoglobin [g/dL]	2 (1.2)	11.2 ± 1.3	10.8 ± 1.5	11.1 ± 1.1	11.6 ± 1.2	< 0.01
Creatinine [mg/dL]	0 (0)	10.4 ± 3.2	10.7 ± 2.8	11.1 ± 3.6	9.6 ± 2.9	0.04
Albumin [g/dL]	0 (0)	3.3 ± 0.5	2.9 ± 0.4	3.4 ± 0.5	3.5 ± 0.3	< 0.01
Phosphorus [mg/dL]	0 (0)	5.4 ± 1.3	5.4 ± 1.2	5.5 ± 1.6	5.4 ± 1.0	0.84
Total Ca [mg/dL]	0 (0)	8.7 ± 0.7	8.5 ± 0.7	8.6 ± 0.6	8.9 ± 0.6	< 0.01
cCa [mg/dL]	0 (0)	9.4 ± 0.7	9.6 ± 0.8	9.3 ± 0.6	9.3 ± 0.6	0.02
iCa [mmol/L]	0 (0)	1.13 ± 0.09	1.09 ± 0.09	1.12 ± 0.07	1.18 ± 0.07	< 0.01
^a^iCa [mg/dL]	0 (0)	4.5 ± 0.4	4.6 ± 0.3	4.4 ± 0.4	4.4 ± 0.4	< 0.01
iCa/cCa ratio [%]	0 (0)	48.2 ± 2.5	45.4 ± 1.5	48.1 ± 0.6	50.7 ± 1.4	< 0.01
Intact PTH [pg/dL]	0 (0)	176 (103–255)	180 (111–269)	177 (121–264)	151 (85–233)	0.54
CRP [mg/dL]	0 (0)	0.12 (0.05–0.36)	0.22 (0.05–0.43)	0.12 (0.05–0.32)	0.08 (0.04–0.23)	0.06

Abbreviations: *PD* peritoneal dialysis, *BMI* body mass index, *CGN* chronic glomerulonephritis, *PCKD* polycystic kidney disease, *CVD* cardiovascular disease, *Ca* calcium, *cCa* corrected calcium, *iCa* ionized calcium, *PTH* parathyroid hormone, *CRP* C-reactive protein

^a^iCa levels represented as mg/dL; these are the same data as above in mmol/L.

PD duration was longer, urine volume and weekly renal Kt/V were lower, and dialysate volume was higher in patients with lower iCa/cCa ratio than in those with higher iCa/cCa ratio. Hemoglobin and serum albumin levels were lower, and CRP tended to be higher in the patients with lower iCa/cCa ratio than in those with higher iCa/cCa ratio.

Total serum Ca and iCa levels were significantly lower, whereas cCa levels and pH were significantly higher in the patients with lower iCa/cCa ratio than in those with higher iCa/cCa ratio. Notably, no between-group differences were observed in terms of serum bicarbonate, phosphorus, and iPTH levels.

### Relationship between the iCa/cCa ratio and RRF

**Fig. 2:**
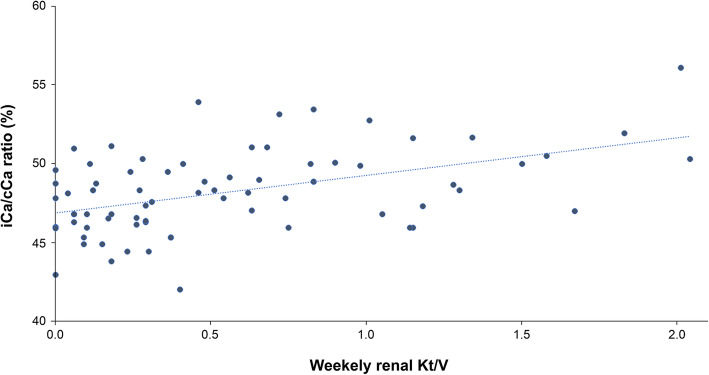
Relationship between iCa/cCa ratio and weekly renal Kt/V. iCa/cCa ratio and weekly renal Kt/V were directly correlated in all 169 study patients (*r =* 0.41, *p* < 0.01). The lower the RRF, the lower the iCa/cCa ratio in the subjects in this study

Figure 2 shows the relationship between iCa/cCa ratio and weekly renal Kt/V. iCa/cCa ratio and weekly renal Kt/V were found to be directly correlated in all study patients (*r =* 0.41, *p* < 0.01).

### Relationship between iCa and cCa in higher and lower RRF groups

**Fig. 3:**
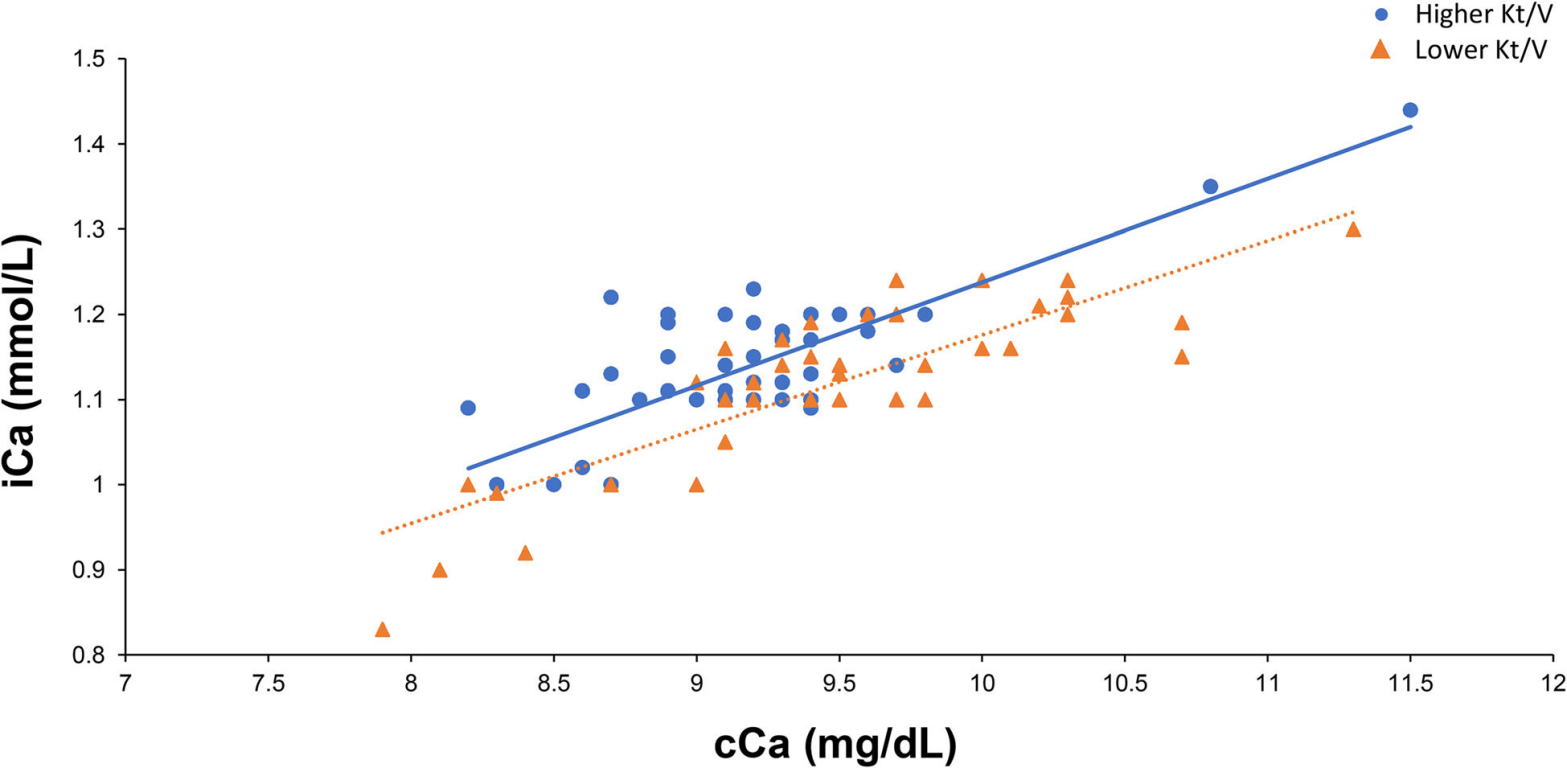
Relationship between iCa and cCa in higher and lower weekly renal Kt/V groups. In both groups, iCa and cCa were directly correlated (*r =* 0.80, *p* < 0.01 and *r =* 0.86, *p <* 0.01 for the higher and lower renal Kt/V groups, respectively). At the same cCa levels, patients with lower RRF had lower iCa levels than those with higher RRF

The relationships between iCa and cCa levels of the higher and lower weekly renal Kt/V groups are shown in Fig. 3. In both groups, iCa and cCa levels were directly correlated (*r =* 0.80, *p <* 0.01 and *r =* 0.86, *p <* 0.01 for higher and lower renal Kt/V groups, respectively). Figure 2 shows that at the same cCa levels, the iCa levels in patients with lower RRF were lower than those in patients with higher RRF.

### Independent factors affecting the relationship between iCa and cCa levels

Table 2 shows the results for multiple regression analysis after multiple imputation, including PD duration, weekly renal Kt/V, pH, hemoglobin levels, creatinine levels, phosphorus levels, and dialysate volume. Among these, weekly renal Kt/V and pH were found to be independent factors affecting iCa/cCa ratio (t = 2.86, *p* < 0.01 and t = − 5.42, *p* < 0.01, respectively). Higher weekly renal Kt/V corresponded to higher iCa/cCa ratio. Furthermore, higher pH corresponded to lower iCa/cCa ratio. We confirmed a similar result that pH and weekly renal Kt/V were independent factors via multiple regression analysis using the data without imputation (Additional file 1). In addition, we selected urinary volume as a surrogate marker of RRF, instead of weekly renal Kt/V, and performed multiple regression analysis with multiple imputation (Additional file 2). Similar results as those for weekly renal Kt/V were obtained; however, the effect of the urinary volume was a tendency (*P* = 0.051) with no significant difference.

**Table 2:** Multiple regression analysis of iCa/cCa ratio

Variable	Regression coefficient	Standard error	t value	*p* value	95% CI
PD duration ^a^	−0.00159	0.00210	−0.75	0.45	−0.00574–0.00257
Renal Kt/V ^a^	0.00579	0.00203	2.86	< 0.01	0.00177–0.00982
pH	−0.246	0.045	−5.42	< 0.01	−0.336−− 0.157
Hemoglobin	0.00149	0.00132	1.13	0.26	−0.00111–0.00410
Creatinine	−0.000558	0.000752	0.46	0.46	−0.000928–0.00205
Phosphate	−0.00305	0.00167	−1.82	0.07	−0.00635–0.000250
Dialysate volume ^a^	−0.00131	0.00547	−0.24	0.81	−0.0121–0.00949

^a^PD duration, weekly renal Kt/V, and dialysate volume are log-transformed

Abbreviation: *PD* peritoneal dialysis

## Discussion

We observed that pH was higher and RRF was lower in PD patients with lower iCa/cCa ratios. Furthermore, pH and RRF were independent factors of iCa/cCa ratio. In PD patients with lower RRF, iCa was lower even when cCa levels were equal to those of patients with maintained RRF, suggesting that the extent of RRF requires consideration in the assessment and management of CKD-MBD in PD patients.

Our results revealed that RRF is an independent factor of iCa/cCa ratio. In patients with low RRF, high volume of PD dialysate could increase pH, possibly contributing to the decreased iCa level. However, dialysis fluid volume was not an independent factor, which may indicate that RRF affects iCa/cCa ratio not only based on dialysate volume but also based on multiple factors, such as elimination of acids.

There have been several reports regarding the importance of RRF in PD patients: RRF contributes to patient survival [21–25] and is associated with cardiovascular diseases [26, 27], nutritional state [28–30], and the incidence of peritonitis [31, 32]. In HD patients, the association between cCa and iCa levels varies; therefore, it is possible to erroneously evaluate ionized hyper- or hypocalcemia by assessing cCa. This has been associated with the risk of death [33]. In patients with lower RRF, the proportion of iCa to cCa levels is lower, and when making clinical judgments based on cCa, the physiologically active iCa level could be inaccurately measured, which can induce secondary hyperparathyroidism, thereby increasing the incidence of cardiovascular diseases. Additionally, it is well known that RRF tends to be better preserved in PD patients than in HD patients after dialysis initiation [34, 35]. Therefore, it could be important to consider RRF for the management of CKD-MBD in PD patients.

In reports regarding RRF and PTH, a higher RRF in HD patients with lower iPTH levels has been reported [36–38]. However, reports on PD patients are few, and there is no consensus on this subject. Although the difference was insignificant, patients with maintained RRF have been reported to have lower iPTH levels [39]. On the other hand, another study has reported that they have higher whole PTH levels [40]. Furthermore, another study has reported that RRF exerts an inhibitive effect on PTH levels [41]. Various factors are involved in these differing PTH levels, one of which could be iCa/cCa ratio. However, the present investigation did not detect any significant differences between iPTH levels in the higher and lower iCa/cCa ratio groups. This may be explained by the effects of pharmacotherapy, including phosphate binders, vitamin D preparations, and Ca-sensing receptor agonists, that could help to adjust PTH to target levels. The present study did not include a very large sample size, and it was impossible to adjust the data for all drugs.

According to the results of this study, pH was another independent factor of iCa/cCa ratio in PD patients. This result was similar to those of previous reports, which stated that iCa levels increased in patients with acidemia and decreased in those with alkalemia [10, 11]. Regarding pH fluctuations, it is necessary to consider not only the metabolic status change but also respiratory compensation. Respiratory compensation could affect pH and consequently change serum iCa concentration. On the other hand, it has been reported that the effect of pH changes on iCa concentration in respiratory changes is blunt compared with that on iCa concentration in metabolic changes [10]. We thus did not monitor pulmonary-related data, such as partial pressure of carbon dioxide, in this study. Additionally, the type of PD dialysate (bicarbonate or bicarbonate/lactate) could have been considered due to its influence on pH; however, we did not have data on the type of PD dialysate.

It is assumed that the Ca concentration of the PD dialysate affects calcium metabolism, and it has been reported that serum Ca concentration decreases by changing Ca concentration from 1.75 mmol/l to 1.25 mmol/l [42, 43]. Furthermore, because many factors affect Ca metabolism in PD patients, including not only PD dialysate Ca concentration but also the degree of RRF, administration of vitamin D, oral administration of Ca-containing P binder, and the amount of diet Ca oral intake, the complexity of Ca balance in PD patients has been reported [44]. In addition, short-term retention and frequent dialysate exchange in anuria APD patients suppress transperitoneal Ca removal has been reported [45]. As a result, we should manage serum Ca concentration in PD patients with the understanding that there are many factors that have an impact on serum Ca concentration. In this study, although these factors could have been considered, not all of them were monitored because the aim of this study was to investigate the factors affecting iCa/cCa ratio and not serum Ca concentration.

This study has several limitations. First, it was a retrospective cross-sectional study, and its retrospective nature prevented us from making claims on causation or their directions. Second, in Japan, the rate of PD in all dialysis patients is low, approximately 3%. Therefore, it was difficult to obtain a large sample size for this study, and our sample size was relatively small (169 patients). Third, because we only evaluated PD patients, it is unknown whether the relationship between RRF and iCa/cCa and pH as well as the iCa/cCa relationship that was observed in PD patients can also be applied to HD patients and non-dialysis CKD patients. Fourth, we did not have the data of a high- or low-calcium dialysate, values of 1, 25-dihydroxy vitamin D and 25-hydroxyvitamin D, and the type of peritoneal transport. Finally, the findings of the present study cannot be reflected in other populations because the distribution of primary kidney disease and several environmental factors differ based on races or regions of the world [46].

## Conclusions

PD patients with a lower iCa/cCa ratio showed higher pH value and reduced RRF. Furthermore, pH and RRF were both independent factors of iCa/cCa ratio. iCa levels were lower in PD patients with reduced RRF, even if their cCa levels were equal to those of patients with maintained RRF. Therefore, for PD patients, the use of cCa alone to perform an assessment of CKD-MBD could result in under- or overestimation of iCa levels, leading to poor management of the disease. CKD-MBD should be cautiously managed on the basis of this difference in daily clinical practice in PD patients for the better management of CKD-MBD as well as for reducing the risk of cardiovascular diseases**.** Further research investigating the mortality and incidence of complications associated with CKD-MBD in large sample sizes are warranted to develop this study’s findings.

## Supplementary information

**Additional file 1.** Multiple regression analysis of iCa/cCa ratio without imputation. Using multiple regression analysis of the data without imputation, we confirmed that pH and weekly renal Kt/V were independent factors affecting iCa/cCa ratio.

**Additional file 2.** Multiple regression analysis of iCa/cCa ratio with multiple imputation, including urinary volume as a surrogate marker of RRF instead of weekly renal Kt/V. pH was an independent factor affecting iCa/cCa ratios. Urinary volume has a tendency to affect iCa/cCa ratio, although it was not significant.

## Data Availability

The datasets used and/or analyzed during the current study are available from the corresponding author upon reasonable request.

## Abbreviations

Alb: Albumin
cCa: Corrected calcium
CKD-MBD: Chronic kidney disease-mineral and bone disorder
HD: Hemodialysis
iCa: Ionized Ca
iPTH: Intact parathyroid hormone
PD: Peritoneal dialysis
PTH: Parathyroid hormone
RRF: Residual renal function
