# Supplementary material for: Factors affecting the relationship between ionized and corrected calcium levels in peritoneal dialysis patients: a retrospective cross-sectional study
Source: BMC Nephrol. 2020 Aug 26;21:370. doi: 10.1186/s12882-020-02033-y (PMC7448483; doi:10.1186/s12882-020-02033-y)
Supplement: Supplementary file 1 — Additional file 1. Multiple regression analysis of iCa/cCa ratio without imputation. Using multiple regression analysis of the data without imputation, we confirmed that pH and weekly renal Kt/V were independent factors affecting iCa/cCa ratio. [file 12882_2020_2033_MOESM1_ESM.docx]

Additional file 1: Multiple regression model for iCa/cCa ratio without imputation

| Variable | Regression coefficient | Standard error | t value | p value | 95% CI |
| --- | --- | --- | --- | --- | --- |
| PD duration ^a^ | −0.00177 | 0.00250 | −0.07 | 0.94 | −0.00514–0.00479 |
| Renal Kt/V ^a^ | 0.00475 | 0.00210 | 2.26 | 0.03 | 0.00058–0.00892 |
| pH | −0.201 | 0.054 | −3.70 | <0.01 | −0.308–−0.093 |
| Hemoglobin | 0.00243 | 0.00152 | 1.60 | 0.11 | −0.00058–0.00544 |
| Creatinine | −0.000486 | 0.000832 | 0.58 | 0.56 | −0.001162–0.002134 |
| Phosphate | −0.00317 | 0.00204 | −1.56 | 0.12 | −0.00721–0.00086 |
| Dialysate volume ^a^ | −0.00774 | 0.00628 | −1.23 | 0.22 | −0.02019–0.00470 |

^a^PD duration, weekly renal Kt/V, and dialysate volume are log transformed.

Abbreviations:

PD: peritoneal dialysis
