# Supplementary material for: Factors affecting the relationship between ionized and corrected calcium levels in peritoneal dialysis patients: a retrospective cross-sectional study
Source: BMC Nephrol. 2020 Aug 26;21:370. doi: 10.1186/s12882-020-02033-y (PMC7448483; doi:10.1186/s12882-020-02033-y)
Supplement: Supplementary file 2 — Additional file 2. Multiple regression analysis of iCa/cCa ratio with multiple imputation, including urinary volume as a surrogate marker of RRF instead of weekly renal Kt/V. pH was an independent factor affecting iCa/cCa ratios. Urinary volume has a tendency to affect iCa/cCa ratio, although it was not significant. [file 12882_2020_2033_MOESM2_ESM.docx]

Additional file 2: Multiple regression analysis of iCa/cCa ratio with imputation including urinary volume

| Variable | Regression coefficient | Standard error | t value | *p* value | 95% CI |
| --- | --- | --- | --- | --- | --- |
| PD duration ^a^ | −0.00280 | 0.0205 | −1.36 | 0.17 | −0.00686–0.00125 |
| Urinary volume ^a^ | 0.00259 | 0.00132 | 1.97 | 0.051 | −0.0000200–0.00520 |
| pH | −0.252 | 0.0460 | −5.47 | <0.01 | −0.342–−0.161 |
| Hemoglobin | 0.00161 | 0.00133 | 1.21 | 0.23 | −0.00102–0.00423 |
| Creatinine | 0.0000270 | 0.000715 | 0.04 | 0.97 | −0.00139–0.00144 |
| Phosphate | −0.00241 | 0.00166 | −1.45 | 0.15 | −0.00568–0.000870 |
| Dialysate volume ^a^ | −0.00393 | 0.00541 | −0.73 | 0.47 | −0.0146–0.00675 |

^a^PD duration, urinary volume, and dialysate volume are log transformed.

Abbreviations: PD, peritoneal dialysis; iCa, active ionized calcium; cCa, corrected calcium.
